# Influence of smoking and other cardiovascular risk factors on heart rate circadian rhythm in normotensive and hypertensive subjects

**DOI:** 10.1371/journal.pone.0257660

**Published:** 2021-09-22

**Authors:** Agostino Accardo, Giulia Silveri, Milos Ajčević, Aleksandar Miladinović, Lorenzo Pascazio

**Affiliations:** 1 Department of Engineering and Architecture, University of Trieste, Trieste, Italy; 2 Department of Medical, Surgical and Health Care, CS of Geriatrics, University of Trieste & ASUGI, Trieste, Italy; Kurume University School of Medicine, JAPAN

## Abstract

Circadian heart rate (HR) is influenced by hypertension and other cardiovascular risk factors particularly smoking, obesity and dyslipidemia. Until now, to evaluate the HR changes due to presence of these risk factors, a single HR office measure or a mean evaluated on day time or night time or 24h was used. However, since HR shows a circadian behavior, a single value represents only a rough approximation of this behavior. In this study, we analyzed the influence of smoking, obesity and dyslipidemia on the circadian rhythm in normotensive and hypertensive subject groups presenting only one of these risk factors. The 24h HR recordings of 170 normotensive (83 without risk factors, 20 smokers, 44 with dyslipidemia, 23 obese) and 353 hypertensive (169 without risk factors, 32 smokers, 99 with dyslipidemia, 53 obese) subjects were acquired using a Holter Blood Pressure Monitor. Results highlighted a specific circadian behavior with three characteristic periods presenting different HR means and rates of HR change in the eight subject groups. The slopes could be used both to estimate the morning HR surge associated with acute cardiovascular effects in the awakening and to evaluate the decline during the night. Moreover, we suggest to use three HR mean values (one for each identified period of the day) rather than two HR values to better describe the circadian HR behavior. Furthermore, smoking increased and dyslipidemia decreased mean HR values from 10:00 to 04:00, both in normotensive and hypertensive subjects in comparison with subjects without risk factors. In this time interval, hypertensive obese subjects showed higher values while normotensive ones presented quite similar values than subjects without risk factors. During the awakening (05:00–10:00) the slopes were similar among all groups with no significant difference among the mean HR values.

## Introduction

Heart rate (HR) is a clinical parameter that reflects the autonomic system activity and reflects the balance of sympathetic and parasympathetic nerves. High values of HR could be associated with endothelial dysfunction that reduce artery compliance and increase arterial wall stress reflecting the decrease of parasympathetic tone and/or the increase of sympathetic tone.

The heart rate measured in office condition is both a potential clinical parameter and a prognostic marker of cardiovascular system due to its association with a wide range of chronic diseases, cardiovascular incidents and all cause of mortality [1,2]. By using office measurement, the Fraingham study [3] identified heart rate as a powerful independent risk factor for cardiovascular morbidity and mortality linking heart rate to myocardial infarction, stroke and death in healthy people [1] as well as in patients with hypertension [4]. In a recent prospective cohort study with 31,507 participants, Wang et al [5] reported that an increase in heart rate by 10 beats/minute was significantly associated with the risk of hypertension. However, conventional office HR measurement has long been used to evaluate physiological states [3] although they do not provide additional prognostic information about short and long term fluctuations during the day [6] as estimated by using the 24h.

On the other hand, the analysis of the HR behavior carried out on the 24h allowed highlighting a decrease of HR values during the night (about 20:00–05:00), high values during the day (about 09:00–19:00) than during the night and an increase during early morning (about 06:00–10:00) [7–11]. However, several studies used the HR mean values calculated only during day time (08:00–21:00) and night time (23:00–06:00) or during the whole 24h, that roughly represent the circadian rhythm, and they proved to be relevant for examining the occurrence of cardiovascular events [12,13]. Moreover, the benefits of using these measurements in the diagnosis and management of hypertension are well known [14]. Nevertheless, the day time, night time as well as the 24h HR mean values are not able to adequately measure what happens in specific periods along 24h such as the quick increment during awakening which is associated with a greater number of episodes of myocardial ischemia, myocardial infarctions and cerebrovascular accidents than to other periods during the day [15].

Furthermore, the presence of cardiovascular risk factors such as smoking, obesity and dyslipidemia affects the sympathetic activity of both normotensive and hypertensive subjects, modifying the HR circadian rhythm [16–23]. In the analysis of a general population stratified by gender and blood pressure levels, Benetos et al [1] found that faster heart rate was associated with a higher overall mortality in both normotensive and hypertensive men. Moreover, they underlined that the association between HR and cardiovascular and coronary deaths was stronger in hypertensive patients. In healthy subjects, the smoking, inducing an acute rise in heart rate in smokers over 15 min after smoking one cigarette [24], is one of the major risk factor for cardiovascular morbidity and mortality. The rise of this clinical parameters seems due to the reduction of the cardiac baroreflex sensitivity of nicotine [25]. Al Safi et al [16], investigating the correlation of smoking with heart rate, highlighted that smokers had significantly higher heart rate, evaluated in office condition, only in male smokers. Bolinder et al [17], studying 135 healthy people, found that office HR values in smokers were significantly lower than the mean values on 24h obtained using ambulatory blood pressure measurement (ABPM). In addition, they found that the mean value of HR during day time was significantly greater in smokers than in non-smokers. On the other hand, in hypertensive patients, Toumilehto et al [18] showed that cardiovascular mortality was three to four times higher in smokers than in non-smokers even if smokers displayed similar heart rate office values than non-smokers. Moreover, Bang et al [19] showed that hypertensive smokers presented significantly higher HR values than non-smokers only during day time with 87±9 beats/min in smokers and 76±11 beats/min in non-smokers. In addition, Verdecchia et al found that HR mean value on 24h was significantly higher in hypertensive smokers than in non-smokers (78±8 beats/min vs 74±9 beats/min) but with near identical office HR values in the two groups (75±10 beats/min vs 74±11 beats/min) [20]. Furthermore, Soresen et al [21] evaluated the impact of smoking on office and ambulatory HR values on treated and non-treated hypertensive subjects, underlining in both groups significantly greater HR values in smokers than in non-smokers during day time (78 ±11 beats/min vs 73±10 beats/min), night time (67±11 beats/min vs 63±10 beats/min) and in office condition (75±13 beats/min vs 72±13 beats/min). In addition, Palatini et al [26], in a cohort of elderly hypertensive subjects, examined the association of clinical and ambulatory HR with total, cardiovascular and non-cardiovascular death. They found that office HR showed a significant positive association with smoking.

Another risk factor is represented by obesity that is considered a potential predictor for cardiovascular disease due to the impairment in the autonomic nervous system controlling HR. In normotensive subjects, Lee et al [22] found higher values of HR in obese than in non-obese subjects in a cohort of 33 subjects highlighting that office HR was 14 beats greater in non-obese group (54 beats/min in vs 40 beats/min). This punctual result was confirmed by Rossi et al [27] that extended the finding also to the mean HR along the 24h in a population of 92 individuals, underling higher mean HR values in obese subjects. This could be due to the fact that in obese subjects the elevated sympathetic activity affected the peripheral vessel and this alteration may be related to the autonomic abnormalities [22,27]. Grassi et al [28] found that in 10 young obese subjects and 8 age matched eutrophic subjects there were no differences in heart rate between the two groups. In addition, Junior et al [29] in 180 obese children between 7–16 years have shown that overweight and obesity are accompanied by higher heart rate values bringing to an alteration in autonomic mechanism. In addition, in a survey studying [23] of 3464 adults with hypertension the authors underlined that obesity was associated with increased heart rate (75±11 beats/min vs 73±10 beats/min), which may at least in part reflect increased arterial stiffness and increased sympathetic tone. Considering the day time and night time, Kotsi et al [30] found in 3216 hypertensive subjects a significant correlation between obesity and mean HR values, with higher values during day time.

About dyslipidemia, Sun et al [31], in a study of 9415 normotensive subjects aged >40 years old, underlined that the cardiovascular risk due to dyslipidemia is related to higher values of office heart rate due to an activation of the sympathetic discharge. In addition, Lee et al [32] in a normotensive population of American Indians underlined that high values of dyslipidemia presented higher values of office HR. In hypertensive subjects Perlini et al. [23] found an increased office heart rate associated with dyslipidemia. Moreover, Palatini et al [26], in elderly hypertensive subjects, found that HR mean values, evaluated along the 24h, showed significant correlations with dyslipidemia.

However, until now the use of a single value calculated either in office or as mean on a long period (day time, night time or 24h) only roughly described the changes due to the circadian rhythm of HR occurring during 24h. Although the latter measurements demonstrated to better evaluate cardiovascular morbidity and mortality in comparison with HR office measure [14], they were not able to adequately measure what happens in specific periods in 24h such as the quick increase during early morning. Moreover, in the literature, the influence of some cardiovascular risk factors was evaluated only considering office or the HR mean values during day time, night time or 24h without considering the circadian rhythm [16–32] and seldom taking into account only one factor at a time.

In order to separately evaluate the influence of specific risk factors (such as: obesity, dyslipidemia and smoking) on the HR circadian rhythm, in this study we carefully examined how the rhythm during 24h changes in normotensive and hypertensive subjects presenting only one risk factor at a time.

The paper extends preliminary results of a previous study [33], in which we analysed only the influence of smoking on the HR circadian rhythm, including subjects presenting more than one risk factor.

## Methods

### Subjects

The study population consisted of 862 subjects afferent to Cardiovascular Pathophysiology (Geriatric Department of the Trieste University&ASUGI, Italy) between June 2016 and September 2016. Based on office BP readings, subjects were classified as hypertensive (SBP≥140 mmHg and/or DBP≥90 mmHg) or non-hypertensive (SBP<140 mmHg and DBP<90 mmHg), according to current Guidelines [34]. Of these, 339 subjects were excluded because they presented evidence of a secondary arterial hypertension or presence of clinical evidence of hypertension related complications. The risk factors we considered were smoking, dyslipidemia and obesity excluding patients suffering from cardiac disease or with type 1 diabetes or presenting more than one risk factor. Thus we considered subjects presenting only one of the examined risk factors. Hence, the cohort of 170 normotensive was composed of 83 subjects without risk factors (N), 20 smokers (N_S_), 44 with dyslipidemia (N_D_) and 23 obese subjects (N_O_). The group of hypertensive patients was formed of 169 patients without risk factors (H), 32 smokers (H_S_), 99 with dyslipidemia (H_D_) and 53 hypertensive with obesity (H_O_). Smokers were defined as those who had smoked for at least a year, obesity was defined as having a body mass index (BMI)>30 [35]. Dyslipidemia is manifested by elevation of the serum total cholesterol (>200 mg/dl), low-density lipoprotein (LDL) cholesterol (>110 mg/dl) and triglyceride concentrations (>150 mg/dl), and a decrease in the high-density lipoprotein (HDL) cholesterol concentration (< 50 mg/dl) [36]. Table 1 reports, separately for all the eight subject groups, the baseline characteristics concerning age and gender. The retrospective study was approved by the institutional review boards (ASUGI Hospital Committee and Regional Ethics Committee). All the subjects gave their written informed consent.

**Table 1:** Mean age (± 1 SD) and gender distribution in the eight subject groups.

	N	N_S_	N_D_	N_O_	H	H_S_	H_D_	H_O_
**Mean Age ±SD (years)**	59±16	54±15	70±11	61±17	64±16	52±14	70±13	65±14
**#Males**	46	13	23	12	95	18	73	34
**#Females**	37	7	21	11	74	14	26	19

### Blood pressure measurement

The BP was measured in office condition, as the average of two consecutive readings [34] and then in ambulatory way, along the 24h, by using a Holter Blood Pressure Monitor (Mobil-O-Graph^®^ NG, IEM gmbh Stolberg, Germany) based on oscillometric technique. The portable monitor was able to record ambulatory blood pressure and heart rate readings each 15-min interval throughout the day (06:00 to 22:00) and each 30-min interval throughout the night (22:00 to 06:00). No patient received additional medication that might affect the circadian heart rate rhythmicity. To examine in detail the circadian trend, the HR values were averaged in each interval separately among all subject of each group. Participants individually received written instructions on how to use the Holter Blood Pressure Monitor. Since in the literature [6,9,19–21], there is no univocal definition of the day and night periods and since the HR profiles showed a specific behavior characterized by three different trends, we chose to subdivide the 24h in three intervals during which we can image that a subject is mostly awake (05:00–10:00), active (10:00–20:00) or slipping (20:00–04:00). In each period, a regression line fitted the linear trend and the linear approximation significance was measured by using the R-square statistic and the p-value. Moreover, the mean and standard deviation values of HR among the subjects was separately examined in each period for each groups. In order to compare our results with those present in the literature, in addition, we evaluated the mean and the standard deviation of HR also during day time (08:00–21:00), night time (23:00–06:00) and along the 24h.

To estimate the influence of each risk factor on HR circadian rhythm in both normotensive and hypertensive subjects, we evaluated separately in each of the three periods, the differences between the HR mean values calculated in N and in each of other normotensive groups (N_S_, N_D_ and N_O_) as well as between H and each of other hypertensive groups. Furthermore, to assess the influence of hypertension on HR rhythm when the same risk factor was present, we estimated the differences between each pair of normotensive and hypertensive corresponding groups (N vs H, N_S_ vs H_S_, etc.). In order to assess these pair comparisons, we applied the Wilcoxon rank sum test with the Bonferroni correction, considering as threshold a P-value of 0.05.

## Results

**Fig 1:**
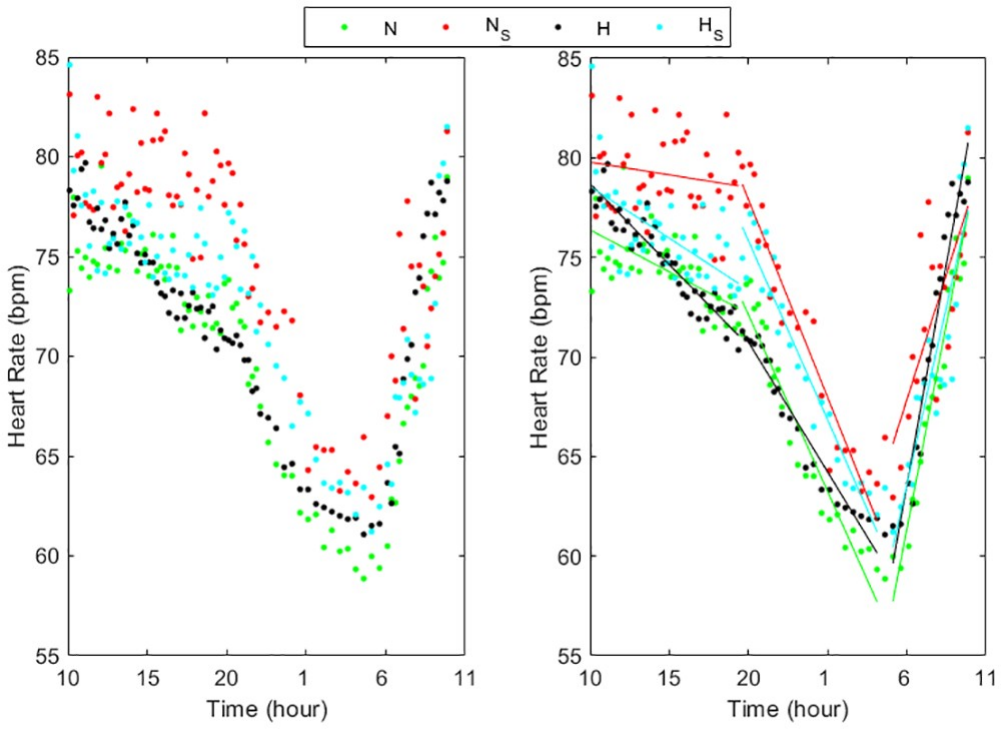
Circadian rhythms of HR in normotensive and hypertensive subjects with and without smoking (left panel) and their linear regression (right panel) in the three periods considered (10:00–20:00, 20:00–4:00, 5:00–10:00). N = Normotensive, N_S_ = Normotensive smoking, H = Hypertensive, H_S_ = Hypertensive smoking.

**Fig 2:**
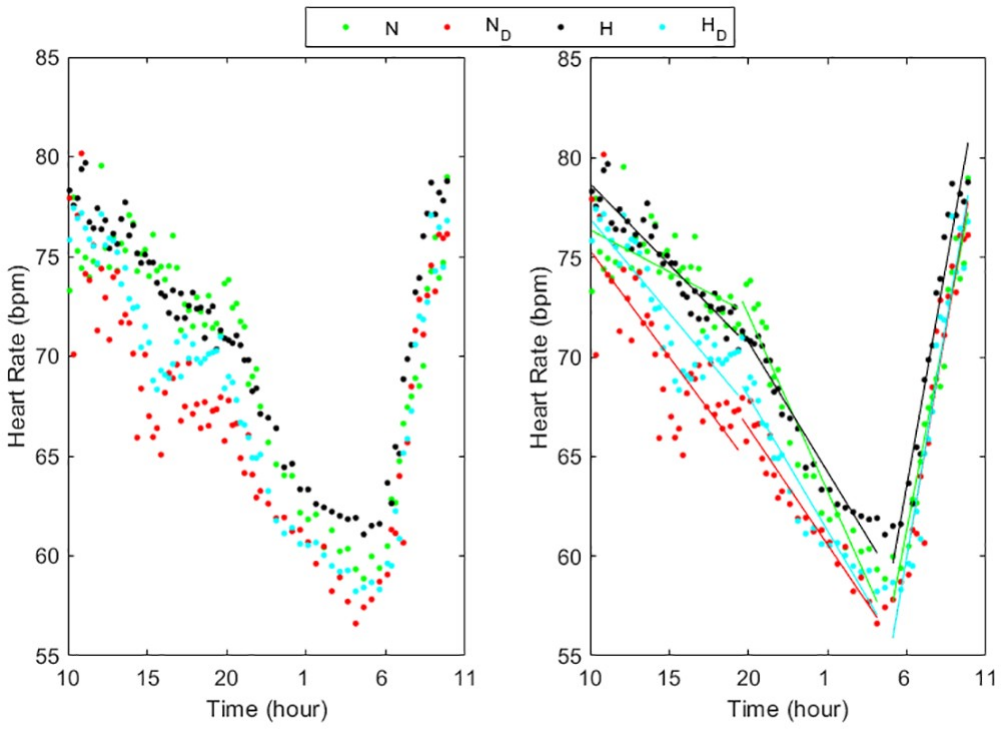
Circadian rhythms of HR in normotensive and hypertensive subjects with and without dyslipidemia (left panel) and their linear regression (right panel) in the three periods considered (10:00–20:00, 20:00–4:00, 5:00–10:00). N = Normotensive, N_D_ = Normotensive with dyslipidemia, H = Hypertensive, H_D_ = Hypertensive with dyslipidemia.

**Fig 3:**
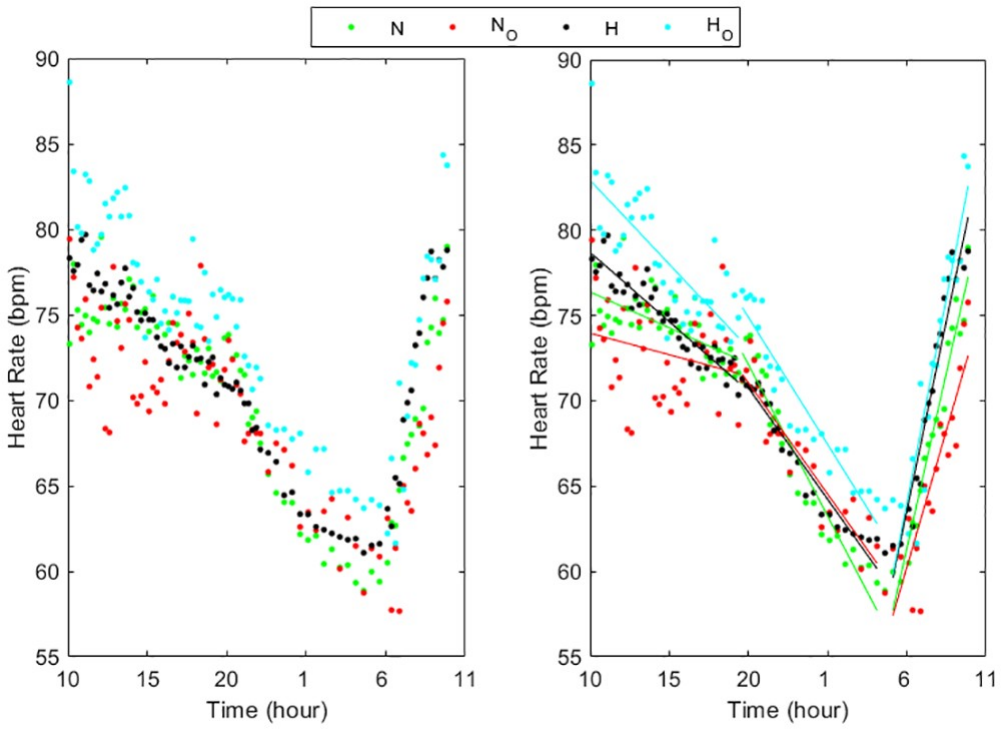
Circadian rhythms of HR in normotensive and hypertensive subjects with and without obesity (left panel) and their linear regression (right panel) in the three periods considered (10:00–20:00, 20:00–4:00, 5:00–10:00). N = Normotensive, N_O_ = Normotensive obese, H = Hypertensive, H_O_ = Hypertensive obese.

Figs 1–3 (left panels) show the circadian rhythms of HR in normotensive and hypertensive subjects with and without each of the three risk factors, separately. All the HR curves decreased moderately between 10:00 and 20:00 in N, N_S_, N_O_ and H_S_ groups while in the other groups there was a larger decrease. Successively, between 20:00 and 4:00 a deeper reduction was present in all groups, less pronounced in N_D_, H, H_D_ and H_O_, followed by an about constant behavior between 04:00 to 05:00 and a quick increase from the early morning till 10:00.

The trend of the HR values in all subject groups can be well approximated in each of the three periods by a linear relation (Figs 1–3, right panels) presenting slopes that differ among groups and periods (Table 2). All the linear relationships showed significant time dependence (p<0.0001) except in the period 10:00–20:00 in smokers and obese normotensive subjects. The high values of the R-squared determination coefficient (Table 2) validated the linear approximations. Between 10:00 and 20:00 the HR slopes were higher in H, H_S_ and H_O_ groups than in the corresponding normotensive groups while in the two groups with dyslipidemia the values were quite similar. From 20:00 to 04:00, the HR slopes in N_S_ groups were higher than in N while in N_D_ and in N_O_ the slopes presented lower values than in N. Also the hypertensive smokers presented in this period higher values of the slope than the other three hypertensive groups. Moreover, the results showed that HR decrease more quickly in smokers during the night. In the early morning, the hypertensive groups presented higher slope values than normotensive subject groups showing that HR increased quicker in the hypertensive patients than in normotensive subjects.

**Table 2:** Slope, intercept and R^2^ values of the linear regression calculated in the three time periods in the eight subject groups.

		N	N_S_	N_D_	N_O_	H	H_S_	H_D_	H_O_
**Slope [bpm/hour]**	**10:00–20:00**	-0.42	-0.13	-1.07	-0.25	-0.81	-0.52	-0.95	-0.99
	**20:00–04:00**	-1.77	-1.99	-1.17	-1.30	-1.30	-1.80	-1.35	-1.49
	**05:00–10:00**	4.12	2.52	4.61	3.21	4.45	3.57	4.68	4.80
**Intercept [bpm]**	**10:00–20:00**	80.6	81.06	86.06	76.48	86.86	83.76	86.43	92.80
	**20:00–04:00**	107.6	117.65	89.92	97.09	96.83	111.80	95.01	104.70
	**05:00–10:00**	-62.04	-7.59	-78.41	-35.92	-69.85	-43.50	-80.50	-79.92
**R square**	**10:00–20:00**	0.63	0.17	0.80	0.25	0.94	0.62	0.88	0.79
	**20:00–04:00**	0.96	0.96	0.97	0.93	0.98	0.97	0.96	0.95
	**05:00–10:00**	0.98	0.75	0.96	0.88	0.98	0.89	0.97	0.93

Table 3 presents the HR mean values (±1SD) for each interval as well as for day time, night time and 24h, in each subject group. In the first period, there were significant differences between each pair of groups except between H vs H_S_ (Table 4). Between 20:00 and 04:00, there were significant differences, close to the limit of significance, only between N vs N_S_, H vs H_D_ and H vs H_O_. Instead, in the awakening, there were no significant differences between the pairs of groups tested. Day time and night time presented behavior similar to that showed during the 10:00–20:00 and the 20:00–04:00 periods, respectively. The means along the 24h shown significant differences as during the day time, except between N and N_O_. Considering the comparison between normal and hypertensive subject groups presenting the same risk factor (Table 5), in the first period, as well as in day time, the differences were statistically significant only when a risk factor (smoking, dyslipidemia or obesity) was present. In the interval 20:00–04:00 no significant difference was found between each pair while during awakening (05:00–10:00) and night time only the difference between N_O_ and H_O_ was significant. Along the 24h the differences between normotensive and hypertensive groups were significant only considering smoking and obesity.

**Table 3:** Mean values ± 1SD of HR values (bpm) in the three periods, during day time, night time and along the 24h, for each subject group.

	N	N_S_	N_D_	N_O_	H	H_S_	H_D_	H_O_
**10:00–20:00**	74±2	79±2	70±4	73±3	75±2	76±2	72±3	78±3
**20:00–04:00**	66±5	71±5	62±3	66±4	66±3	70±5	63±3	70±4
**05:00–10:00**	68±6	72±5	68±7	66±5	71±6	70±6	68±7	72±7
**Day time (08:00–21:00)**	74±2	78±3	70±4	72±3	75±3	76±3	72±3	78±3
**Night time (23:00–06:00)**	61±2	66±3	59±2	63±2	63±1	65±3	60±1	66±2
**24h**	71±6	75±5	67±6	69±5	71±5	73±5	69±6	74±6

**Table 4:** P-value of the comparison between normotensive and hypertensive groups with and without risk factors.

	N vs N_S_	N vs N_D_	N vs N_O_	H vs H_S_	H vs H_D_	H vs H_O_
**10:00–20:00**	p<0.0006 Z>3.4	p<0.0006 Z>3.4	p = 0.018 Z = 2.36	n.s.	p = 0.0024 Z = 3.04	p<0.0006 Z>3.4
**20:00–04:00**	p = 0.024 Z = 2.25	n.s.	n.s.	n.s.	p = 0.036 Z = 2.1	p = 0.042 Z = 2.03
**05:00–10:00**	n.s.	n.s.	n.s.	n.s.	n.s.	n.s.
**Day time (08:00–21:00)**	p<0.00006 Z>4.01	p<0.00006 Z>4.01	p = 0.0024 Z = 3.04	n.s.	p<0.0006 Z>3.4	p<0.0006 Z>3.4
**Night time (23:00–06:00)**	p<0.00006 Z>4.01	n.s.	n.s.	n.s.	p<0.0006 Z>3.4	p = 0.0024 Z = 3.04
**24h**	p<0.00006 Z>4.01	p = 0.0012 Z = 3.25	n.s.	n.s.	p = 0.009 Z = 2.61	p = 0.0102 Z = 2.58

**Table 5:** P-value of the comparison between normotensive and hypertensive groups presenting the same risk factor.

	N vs H	N_S_ vs H_S_	N_D_ vs H_D_	N_O_ vs H_O_
**10:00–20:00**	n.s.	p<0.0004 Z>3.54	0.006	p<0.0004 Z>3.54
**20:00–04:00**	n.s.	n.s.	n.s.	n.s.
**05:00–10:00**	n.s.	n.s.	n.s.	p = 0.028 Z = 2.2
**Day time (08:00–21:00)**	n.s.	p<0.00004 Z>4.1	p = 0.0024 Z = 3.04	p<0.00004 Z>4.1
**Night time (23:00–06:00)**	n.s.	n.s.	n.s.	p = 0.012 Z = 2.51
**24h**	n.s.	p = 0.0012 Z = 3.25	n.s.	p<0.00004 Z>4.1

## Discussion

Since a punctual measurement of HR, like the office measure or the mean on day, night or 24h, might be poorly representative of the HR rhythm, leading to a reduction of the real predictive power of HR analysis, and since various risk factors influence the circadian rhythm of HR, in our work we examined in detail how this rhythm changes during 24h in normotensive and hypertensive subjects presenting only one risk factor at a time. In particular, in the eight subject groups concerning normotensive and hypertensive subjects either without or with one risk factor, this rhythm showed a quite peculiar and different behavior on three specific time periods, with different linear trends among groups (Figs 1–3) similar to that found in a previous our preliminary study [33] concerning normotensive and hypertensive subjects without distinguishing the risk factors. Consequently, we separately analyzed every HR trend during each of these three intervals. The circadian profiles in all the groups showed a HR decrease during the first period, mainly diurnal (10:00 to 20:00), followed by a quicker decrease in the second period, mainly nocturnal (20:00 to 04:00), and a remarkable increase during the awakening (05:00 to 10:00) till to reach a maximum HR value at about 10:00, inducing elevations in arterial stiffness that could be the cause of cerebrovascular accidents [1,4]. The first and the second period presented a trend opposed to that between 05:00 and 10:00, similarly to that already known in the literature regarding the HR differences between day time (quite overlapping our first period) and night time (partially overlapping our second and third period), associated to sleep [11]. Moreover, we underlined a rapid rise in heart rate in the early morning, as reported in [10,11,13] in the period from 06:00–08:00, probably due to the response when going from a supine to a standing position [10] also reflecting an increase in sympathetic nervous system [13]. Furthermore, our results extended the period from 05:00 to 10:00, probably because of the preparatory mechanism for awakening by the autonomous system which provide the indispensable increase in general vasoconstrictor tone and in part to the adaptation to the activities carried out in the early morning until a maximum value at about 10:00.

In order to quantify the velocity at which the heart rate changes in the three periods and the influence of the risk factors on these changes, we estimated the slopes of the linear approximation in each interval, highlighting different rates among subject groups and the interval of the day (Tab.2, Figs 1–3). During the first and third period (day time and early morning) hypertensive without other risk factors as well as smokers or presenting obesity showed greater velocity change than corresponding normotensive subjects. In the second period (night time) the differences between the slopes in H_O_ and in N_O_ as well as between H_S_ and N_S_ patients became similar, while H patients showed lower slopes than N subjects. In subjects affected by dyslipidemia, the differences between normotensive and hypertensive were negligible in all the three periods. The slopes we calculated in the third period could be used to quantify the morning HR surge associated with acute cardiovascular effects while the values calculated from 20:00 to 04:00 could be used to measure the decline during night. The different slopes could indicate different control ways of the HR changes along the 24h depending on the risk factor. Its specific meaning is not known but greater slopes could mean the need of a cardiac compensation that is difficult to achieve and therefore requires greater strain on the myocardium. At the same time, a lower slope could mean an inability of the myocardium to reach efficient responses.

Considering the mean HR values, the normotensive group without risk factors presented greater values between 10:00 and 20:00 than in the other two periods, confirming the results of Casiglia [9]. Moreover, our results (Tables 3 and 4) highlighted that all risk factors considered (smoking, obesity and dyslipidemia) affected the mean HR values with a significant variation during the day (10:00–20:00 as well as 08:00–21:00). In particular, in that period the normotensive smokers presented the highest HR values, the normotensive subjects with dyslipidemia the lowest and those with obesity intermediate values. During the night (20:00–04:00 as well as 23:00–06:00) only smokers presented a significant difference compared to normotensive without risk factors (Tables 3 and 4). These results confirmed the findings of [19] in which greater HR values in smokers during day time and night time were found, probably because of the sympathomimetic effects of nicotine and the worse oxygen exchange in the lugs of smokers that increased myocardial oxygen consumption at rest. In relation to obesity, the difference during the day confirmed the results of [22,27] underlying a sympathetic predominance and a reduction in parasympathetic activity in that period of the day in these subjects. Moreover, the lower HR values in subjects with dyslipidemia maybe due to a reduced activation of sympathetic stimulation partially confirming the results of [31,32]. As expected, during the 24h the mean values showed an intermediate behavior in comparison with day time and night time as reported by [15]. We would underline that, in comparison with the values averaged on day time, night time or on 24h, our approach allowed to highlight that during the last period (05:00–10:00) the slopes were quite similar and the differences of mean HR due to each risk factor were not significant.

Considering the four hypertensive groups, the smoking does not affect significantly the HR mean values in any of the three periods (Tables 3 and 4), confirming the results of [20,21] obtained in day time and night time. This may be due to the fact that the smoke imposes on the heart an increase in cardiac oxygen consumption [20]. In addition, hypertensive patients with obesity showed statistically higher HR values during all the three periods than hypertensive without risk factors, confirming the results of [23,30] obtained on patients affected by metabolic syndrome during both day time and night time and in office condition. This behavior could be due to the increase of arterial stiffness and sympathetic tone present in obese subjects. Moreover, our results suggested that the presence of dyslipidemia reduce the HR values during all the day, significantly from 10:00 to 04:00, extending the results of [31,32] in which only office measurements were considered. This reduction of HR was probably due to the reduced activation of sympathetic nervous system. For hypertensive patients, the mean HR values calculated on day time were comparable to those evaluated during the first period (10:00–20:00) while the values estimated on 24h as well as on night time presented intermediated values between those of the second and third periods (20:00–04:00, 05:00–10:00). Hence, our analysis allowed to better examine the HR behavior in these two periods in which the trends were opposite highlighting a significant difference between the mean values of H_O_ and H and of H_D_ and H between 20:00 and 04:00.

Comparing hypertensive and normotensive subjects considering the same risk factor, our results showed no significant differences between N and H in the three periods, confirming the findings of [15] obtained during day time and night time and 24h, suggesting a primary role of central nervous mechanisms in both subject groups and also highlighting that the marked impairment of the arterial baroreceptor did not modify HR variabilities. Between H_S_ e N_S_ and N_D_ and H_D_ the differences were statistically significant between 10:00 and 20:00 as well as during day time. The mean HR values on 24h were mainly affected by the HR values during day time in H_S_ group and by the HR values during the night time in H_D_ group. Finally, considering the obesity, there were significant differences only between 05:00 and 20:00 as well as during day time. The night time mean values were significantly different, probably because of the behavior between 04:00 and 06:00.

## Conclusion

In conclusion, in comparison with the literature in which the HR circadian rhythm is segmented in only two part (day time and night time) our analysis allowed describing in a more precise way the changes in the rhythm identifying three periods, each presenting a specific linear trend. The slopes in normotensive and hypertensive subjects with and without risk factors, showed different values highlighting different velocity rates of HR changes among the considered groups and periods that probably may be associated with different risk levels of cardiovascular disease. However, links with clinical outcomes are needed to confirm this hypothesis. In any case we suggest at least the use of three mean HR values (one for each period) instead of the two values currently used that roughly described the rhythm. About the influence of some risk factor on the circadian rhythm, our results highlighted that in the first two periods (from 10:00 to 04:00) smokers presented higher HR values and subjects with dyslipidemia lower values than subjects without risk factors. Concerning obesity, hypertensive subjects showed higher values while normotensive ones presented quite similar values than subjects without risk factors. During the awakening (05:00–10:00) the slopes were similar among all groups with no significant difference among the mean HR values.
